# 3-Methyl-5-phen­oxy-1-phenyl-1*H*-pyrazole-4-carbaldehyde

**DOI:** 10.1107/S1600536811036786

**Published:** 2011-09-14

**Authors:** Tara Shahani, Hoong-Kun Fun, Shobhitha Shetty, Balakrishna Kalluraya

**Affiliations:** a X-ray Crystallography Unit, School of Physics, Universiti Sains Malaysia, 11800 USM, Penang, Malaysia; b Department of Studies in Chemistry, Mangalore University, Mangalagangotri, Mangalore 574 199, India

## Abstract

In the title compound, C_17_H_14_N_2_O_2_, the pyrazole ring makes dihedral angles of 73.67 (4) and 45.99 (4)°, respectively, with the adjacent phenyl and phen­oxy rings. In the crystal, there are no classical hydrogen bonds, but a weak C—H⋯π inter­action is observed.

## Related literature

For biological applications of pyrazole derivatives, see: Rai *et al.* (2008 ▶); Isloor *et al.* (2009 ▶); Girisha *et al.* (2010 ▶). For a related structure, see: Shahani *et al.* (2011 ▶). For bond-length data, see: Allen *et al.* (1987 ▶).
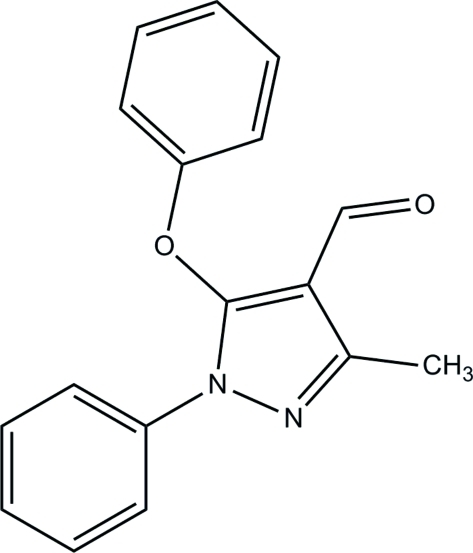


## Experimental

### 

#### Crystal data

C_17_H_14_N_2_O_2_
*M* *_r_* = 278.30
Monoclinic, 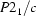

*a* = 8.6207 (1) Å
*b* = 7.1695 (1) Å
*c* = 22.9228 (3) Å
β = 99.168 (1)°
*V* = 1398.67 (3) Å^3^
*Z* = 4
Mo *K*α radiation
μ = 0.09 mm^−1^
*T* = 100 K
0.46 × 0.20 × 0.14 mm

#### Data collection

Bruker SMART APEXII CCD area-detector diffractometer
Absorption correction: multi-scan (*SADABS*; Bruker, 2009 ▶) *T*_min_ = 0.961, *T*_max_ = 0.988
24894 measured reflections
6610 independent reflections
5063 reflections with *I* > 2σ(*I*)
*R*_int_ = 0.035

#### Refinement

*R*[*F* ^2^ > 2σ(*F* ^2^)] = 0.044
*wR*(*F* ^2^) = 0.127
*S* = 1.06
6610 reflections
191 parameters
H-atom parameters constrained
Δρ_max_ = 0.49 e Å^−3^
Δρ_min_ = −0.25 e Å^−3^

### 

Data collection: *APEX2* (Bruker, 2009 ▶); cell refinement: *SAINT* (Bruker, 2009 ▶); data reduction: *SAINT*; program(s) used to solve structure: *SHELXTL* (Sheldrick, 2008 ▶); program(s) used to refine structure: *SHELXTL*; molecular graphics: *SHELXTL*; software used to prepare material for publication: *SHELXTL* and *PLATON* (Spek, 2009 ▶).

## Supplementary Material

Crystal structure: contains datablock(s) global, I. DOI: 10.1107/S1600536811036786/is2775sup1.cif

Structure factors: contains datablock(s) I. DOI: 10.1107/S1600536811036786/is2775Isup2.hkl

Supplementary material file. DOI: 10.1107/S1600536811036786/is2775Isup3.cml

Additional supplementary materials:  crystallographic information; 3D view; checkCIF report

## Figures and Tables

**Table 1:** Hydrogen-bond geometry (Å, °) *Cg*1 is the centroid of the C1–C6 ring.

*D*—H⋯*A*	*D*—H	H⋯*A*	*D*⋯*A*	*D*—H⋯*A*
C11—H11*A*⋯*Cg*1^i^	0.95	2.62	3.5052 (8)	156

Symmetry code: (i) 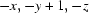.

**Fig. 1.**
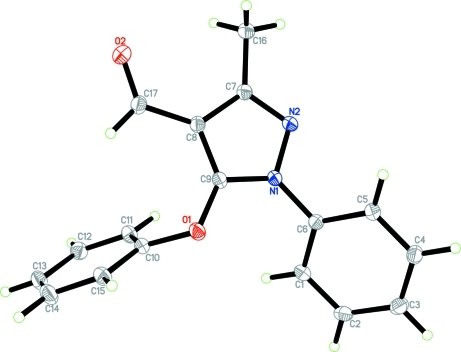
The molecular structure of the title compound, showing 30% probability displacement ellipsoids and the atom-numbering scheme.

**Fig. 2.**
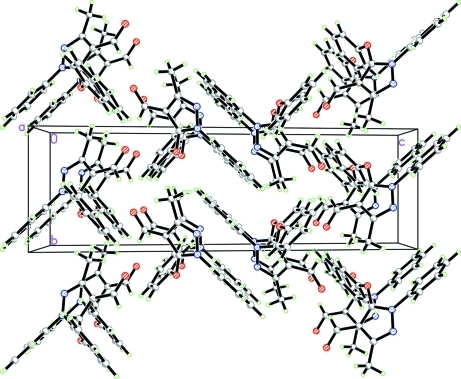
The crystal packing of the title compound, viewed along the a axis.

## supplementary crystallographic information

### Comment

Pyrazoles are a novel class of heterocyclic compounds possessing wide variety of application in the agrochemical and pharmaceutical industries. Derivatives of pyrazoles are found to show good antibacterial (Rai *et al.*, 2008), anti-inflammatory and analgesic (Isloor *et al.*, 2009) activities. In view of these observations and in continuation of our search for biologically active pyrazole derivatives, we herein report the crystal structure of 3-methyl-5-phenoxy-1-phenyl-1*H*-pyrazole-4-carbaldehyde. Reaction of 5-chloro-3-methyl-1-phenyl-1*H*-pyrazole-4-carbaldehyde with phenol afforded 5-chloro-3-methyl-1-phenyl-1*H*-pyrazole-4-carbaldehyde (Girisha *et al.*, 2010).

The asymmetric unit of the title compound is shown in Fig. 1. The 1*H*-pyrazole (N1/N2/C7–C9) ring is essentially planar with a maximum deviation of 0.004 (1) Å for atom N1. The central pyrazole ring makes dihedral angles of 73.67 (4) and 45.99 (4)° with the terminal phenyl (C1–C6) and (C10–C15) rings, respectively. The bond lengths (Allen *et al.*, 1987) and angles are within normal ranges and is comparable to a closely related structure (Shahani *et al.*, 2011).

In the crystal packing (Fig. 2), there are no classical hydrogen bonds but stabilization is provided by weak C—H···π (Table 1) interactions, involving the centroid *Cg*1 of the C1–C6 ring.

### Experimental

5-Chloro-3-methyl-1-phenyl-1*H*-pyrazol-4-carboxaldehyde (0.1 mol) and phenol (0.1 mol) was dissolved in 10 mL of dimethyl sulfoxide. To this solution, 5.6 g (0.1 mol) of potassium hydroxide was added. The reaction mixture was refluxed for 6 hrs and then was cooled to room temperature and poured to crushed ice. The solid product that separated was filtered and dried. It was then recrystallized from ethanol. Crystals suitable for X-ray analysis were obtained from 1:2 mixtures of DMF and ethanol by slow evaporation.

### Refinement

All the H atoms were positioned geometrically (C—H = 0.95–0.98 Å) and were refined using a riding model, with *U*_iso_(H) =1.2 or 1.5*U*_eq_(C).

### Crystal data

**Table:**

C_17_H_14_N_2_O_2_	*F*(000) = 584
*M**_r_* = 278.30	*D*_x_ = 1.322 Mg m^−^^3^
Monoclinic, *P*2_1_/*c*	Mo *K*α radiation, λ = 0.71073 Å
Hall symbol: -P 2ybc	Cell parameters from 7046 reflections
*a* = 8.6207 (1) Å	θ = 3.7–36.0°
*b* = 7.1695 (1) Å	µ = 0.09 mm^−^^1^
*c* = 22.9228 (3) Å	*T* = 100 K
β = 99.168 (1)°	Block, colourless
*V* = 1398.67 (3) Å^3^	0.46 × 0.20 × 0.14 mm
*Z* = 4	

### Data collection

**Table:**

Bruker SMART APEXII CCD area-detector diffractometer	6610 independent reflections
Radiation source: fine-focus sealed tube	5063 reflections with *I* > 2σ(*I*)
graphite	*R*_int_ = 0.035
φ and ω scans	θ_max_ = 36.1°, θ_min_ = 2.8°
Absorption correction: multi-scan (*SADABS*; Bruker, 2009)	*h* = −14→14
*T*_min_ = 0.961, *T*_max_ = 0.988	*k* = −10→11
24894 measured reflections	*l* = −37→37

### Refinement

**Table:**

Refinement on *F*^2^	Primary atom site location: structure-invariant direct methods
Least-squares matrix: full	Secondary atom site location: difference Fourier map
*R*[*F*^2^ > 2σ(*F*^2^)] = 0.044	Hydrogen site location: inferred from neighbouring sites
*wR*(*F*^2^) = 0.127	H-atom parameters constrained
*S* = 1.06	*w* = 1/[σ^2^(*F*_o_^2^) + (0.0667*P*)^2^ + 0.1411*P*] where *P* = (*F*_o_^2^ + 2*F*_c_^2^)/3
6610 reflections	(Δ/σ)_max_ = 0.001
191 parameters	Δρ_max_ = 0.49 e Å^−^^3^
0 restraints	Δρ_min_ = −0.25 e Å^−^^3^

### Special details

**Table:**

Geometry. All e.s.d.'s (except the e.s.d. in the dihedral angle between two l.s. planes) are estimated using the full covariance matrix. The cell e.s.d.'s are taken into account individually in the estimation of e.s.d.'s in distances, angles and torsion angles; correlations between e.s.d.'s in cell parameters are only used when they are defined by crystal symmetry. An approximate (isotropic) treatment of cell e.s.d.'s is used for estimating e.s.d.'s involving l.s. planes.
Refinement. Refinement of *F*^2^ against ALL reflections. The weighted *R*-factor *wR* and goodness of fit *S* are based on *F*^2^, conventional *R*-factors *R* are based on *F*, with *F* set to zero for negative *F*^2^. The threshold expression of *F*^2^ > σ(*F*^2^) is used only for calculating *R*-factors(gt) *etc*. and is not relevant to the choice of reflections for refinement. *R*-factors based on *F*^2^ are statistically about twice as large as those based on *F*, and *R*- factors based on ALL data will be even larger.

### Fractional atomic coordinates and isotropic or equivalent isotropic displacement parameters (Å^2^)

**Table:**

	*x*	*y*	*z*	*U*_iso_*/*U*_eq_	
O1	0.05681 (7)	0.69565 (8)	0.13280 (3)	0.01742 (12)	
O2	0.06804 (8)	0.18778 (10)	0.24152 (3)	0.02350 (14)	
N1	−0.13873 (7)	0.51835 (10)	0.07691 (3)	0.01459 (12)	
N2	−0.21777 (8)	0.35085 (10)	0.07780 (3)	0.01602 (12)	
C1	−0.08495 (9)	0.74369 (12)	0.00220 (3)	0.01637 (14)	
H1A	0.0246	0.7199	0.0118	0.020*	
C2	−0.14204 (10)	0.87209 (12)	−0.04165 (3)	0.01873 (15)	
H2A	−0.0707	0.9369	−0.0620	0.022*	
C3	−0.30261 (10)	0.90625 (12)	−0.05596 (3)	0.02026 (15)	
H3A	−0.3404	0.9951	−0.0856	0.024*	
C4	−0.40764 (10)	0.81003 (12)	−0.02678 (3)	0.01878 (15)	
H4A	−0.5173	0.8318	−0.0370	0.023*	
C5	−0.35271 (9)	0.68226 (12)	0.01719 (3)	0.01632 (14)	
H5A	−0.4243	0.6168	0.0372	0.020*	
C6	−0.19152 (9)	0.65081 (11)	0.03172 (3)	0.01421 (13)	
C7	−0.14856 (9)	0.26348 (11)	0.12608 (3)	0.01569 (13)	
C8	−0.02490 (8)	0.37265 (11)	0.15780 (3)	0.01490 (13)	
C9	−0.02436 (8)	0.53407 (11)	0.12425 (3)	0.01441 (13)	
C10	0.22016 (9)	0.68417 (11)	0.15060 (3)	0.01485 (13)	
C11	0.30911 (9)	0.55321 (12)	0.12616 (3)	0.01733 (14)	
H11A	0.2605	0.4651	0.0981	0.021*	
C12	0.47161 (9)	0.55367 (13)	0.14369 (3)	0.01931 (15)	
H12A	0.5342	0.4630	0.1281	0.023*	
C13	0.54285 (10)	0.68545 (13)	0.18366 (4)	0.02145 (16)	
H13A	0.6538	0.6863	0.1949	0.026*	
C14	0.45058 (10)	0.81631 (13)	0.20720 (4)	0.02241 (17)	
H14A	0.4992	0.9067	0.2344	0.027*	
C15	0.28718 (10)	0.81597 (12)	0.19112 (3)	0.01855 (15)	
H15A	0.2238	0.9038	0.2075	0.022*	
C16	−0.20510 (10)	0.07608 (13)	0.14161 (4)	0.02157 (16)	
H16A	−0.2763	0.0253	0.1078	0.032*	
H16B	−0.2610	0.0877	0.1755	0.032*	
H16C	−0.1151	−0.0078	0.1518	0.032*	
C17	0.07517 (9)	0.33089 (12)	0.21332 (3)	0.01724 (14)	
H17A	0.1514	0.4212	0.2288	0.021*	

### Atomic displacement parameters (Å^2^)

**Table:**

	*U*^11^	*U*^22^	*U*^33^	*U*^12^	*U*^13^	*U*^23^
O1	0.0151 (2)	0.0131 (3)	0.0227 (2)	−0.00067 (19)	−0.00134 (19)	−0.0014 (2)
O2	0.0227 (3)	0.0224 (3)	0.0238 (3)	−0.0006 (2)	−0.0010 (2)	0.0070 (2)
N1	0.0127 (2)	0.0135 (3)	0.0167 (2)	−0.0012 (2)	−0.00011 (19)	0.0005 (2)
N2	0.0139 (3)	0.0141 (3)	0.0194 (3)	−0.0023 (2)	0.0007 (2)	0.0013 (2)
C1	0.0155 (3)	0.0157 (3)	0.0180 (3)	−0.0027 (2)	0.0028 (2)	−0.0014 (2)
C2	0.0222 (3)	0.0163 (4)	0.0179 (3)	−0.0049 (3)	0.0038 (3)	0.0003 (3)
C3	0.0251 (4)	0.0160 (4)	0.0184 (3)	−0.0009 (3)	−0.0004 (3)	0.0022 (3)
C4	0.0173 (3)	0.0180 (4)	0.0197 (3)	0.0016 (3)	−0.0012 (3)	0.0006 (3)
C5	0.0142 (3)	0.0176 (4)	0.0169 (3)	−0.0006 (2)	0.0015 (2)	0.0008 (2)
C6	0.0143 (3)	0.0136 (3)	0.0144 (3)	−0.0003 (2)	0.0013 (2)	0.0000 (2)
C7	0.0135 (3)	0.0149 (3)	0.0183 (3)	0.0003 (2)	0.0017 (2)	0.0013 (2)
C8	0.0136 (3)	0.0146 (3)	0.0160 (3)	0.0003 (2)	0.0008 (2)	0.0001 (2)
C9	0.0122 (3)	0.0143 (3)	0.0162 (3)	−0.0003 (2)	0.0007 (2)	−0.0018 (2)
C10	0.0138 (3)	0.0150 (3)	0.0151 (3)	−0.0018 (2)	0.0004 (2)	0.0004 (2)
C11	0.0165 (3)	0.0183 (4)	0.0168 (3)	−0.0016 (3)	0.0018 (2)	−0.0028 (3)
C12	0.0166 (3)	0.0222 (4)	0.0196 (3)	−0.0003 (3)	0.0044 (2)	0.0003 (3)
C13	0.0154 (3)	0.0261 (4)	0.0220 (3)	−0.0046 (3)	0.0003 (3)	0.0010 (3)
C14	0.0207 (4)	0.0236 (4)	0.0215 (3)	−0.0067 (3)	−0.0009 (3)	−0.0043 (3)
C15	0.0190 (3)	0.0175 (4)	0.0186 (3)	−0.0034 (3)	0.0014 (2)	−0.0036 (3)
C16	0.0189 (3)	0.0168 (4)	0.0274 (3)	−0.0031 (3)	−0.0011 (3)	0.0052 (3)
C17	0.0160 (3)	0.0179 (4)	0.0170 (3)	0.0013 (3)	0.0001 (2)	0.0007 (2)

### Geometric parameters (Å, °)

**Table:**

O1—C9	1.3514 (10)	C7—C16	1.4916 (12)
O1—C10	1.4047 (9)	C8—C9	1.3899 (11)
O2—C17	1.2194 (10)	C8—C17	1.4505 (10)
N1—C9	1.3496 (9)	C10—C15	1.3850 (11)
N1—N2	1.3825 (10)	C10—C11	1.3858 (11)
N1—C6	1.4254 (10)	C11—C12	1.3945 (11)
N2—C7	1.3276 (10)	C11—H11A	0.9500
C1—C2	1.3937 (11)	C12—C13	1.3892 (12)
C1—C6	1.3942 (10)	C12—H12A	0.9500
C1—H1A	0.9500	C13—C14	1.3931 (13)
C2—C3	1.3925 (12)	C13—H13A	0.9500
C2—H2A	0.9500	C14—C15	1.3981 (12)
C3—C4	1.3914 (12)	C14—H14A	0.9500
C3—H3A	0.9500	C15—H15A	0.9500
C4—C5	1.3882 (11)	C16—H16A	0.9800
C4—H4A	0.9500	C16—H16B	0.9800
C5—C6	1.3947 (10)	C16—H16C	0.9800
C5—H5A	0.9500	C17—H17A	0.9500
C7—C8	1.4250 (11)		
			
C9—O1—C10	117.63 (6)	N1—C9—C8	107.99 (7)
C9—N1—N2	111.06 (6)	O1—C9—C8	132.91 (7)
C9—N1—C6	129.53 (7)	C15—C10—C11	122.33 (7)
N2—N1—C6	119.24 (6)	C15—C10—O1	116.53 (7)
C7—N2—N1	105.37 (6)	C11—C10—O1	121.06 (7)
C2—C1—C6	118.77 (7)	C10—C11—C12	118.50 (7)
C2—C1—H1A	120.6	C10—C11—H11A	120.8
C6—C1—H1A	120.6	C12—C11—H11A	120.8
C3—C2—C1	120.64 (7)	C13—C12—C11	120.65 (8)
C3—C2—H2A	119.7	C13—C12—H12A	119.7
C1—C2—H2A	119.7	C11—C12—H12A	119.7
C4—C3—C2	119.88 (7)	C12—C13—C14	119.59 (8)
C4—C3—H3A	120.1	C12—C13—H13A	120.2
C2—C3—H3A	120.1	C14—C13—H13A	120.2
C5—C4—C3	120.22 (7)	C13—C14—C15	120.68 (8)
C5—C4—H4A	119.9	C13—C14—H14A	119.7
C3—C4—H4A	119.9	C15—C14—H14A	119.7
C4—C5—C6	119.46 (7)	C10—C15—C14	118.23 (8)
C4—C5—H5A	120.3	C10—C15—H15A	120.9
C6—C5—H5A	120.3	C14—C15—H15A	120.9
C1—C6—C5	121.02 (7)	C7—C16—H16A	109.5
C1—C6—N1	120.81 (7)	C7—C16—H16B	109.5
C5—C6—N1	118.16 (7)	H16A—C16—H16B	109.5
N2—C7—C8	111.50 (7)	C7—C16—H16C	109.5
N2—C7—C16	120.23 (7)	H16A—C16—H16C	109.5
C8—C7—C16	128.27 (7)	H16B—C16—H16C	109.5
C9—C8—C7	104.08 (6)	O2—C17—C8	124.45 (8)
C9—C8—C17	127.11 (7)	O2—C17—H17A	117.8
C7—C8—C17	128.78 (7)	C8—C17—H17A	117.8
N1—C9—O1	118.89 (7)		
			
C9—N1—N2—C7	−0.74 (8)	C6—N1—C9—O1	0.07 (12)
C6—N1—N2—C7	−176.34 (6)	N2—N1—C9—C8	0.53 (9)
C6—C1—C2—C3	−0.36 (12)	C6—N1—C9—C8	175.55 (7)
C1—C2—C3—C4	−0.75 (12)	C10—O1—C9—N1	−137.81 (7)
C2—C3—C4—C5	1.03 (12)	C10—O1—C9—C8	48.05 (11)
C3—C4—C5—C6	−0.20 (12)	C7—C8—C9—N1	−0.11 (8)
C2—C1—C6—C5	1.21 (11)	C17—C8—C9—N1	−178.21 (7)
C2—C1—C6—N1	−179.97 (7)	C7—C8—C9—O1	174.49 (8)
C4—C5—C6—C1	−0.93 (12)	C17—C8—C9—O1	−3.61 (14)
C4—C5—C6—N1	−179.79 (7)	C9—O1—C10—C15	−142.16 (7)
C9—N1—C6—C1	49.63 (11)	C9—O1—C10—C11	41.12 (10)
N2—N1—C6—C1	−135.69 (8)	C15—C10—C11—C12	0.73 (12)
C9—N1—C6—C5	−131.51 (8)	O1—C10—C11—C12	177.26 (7)
N2—N1—C6—C5	43.17 (10)	C10—C11—C12—C13	−1.55 (12)
N1—N2—C7—C8	0.67 (9)	C11—C12—C13—C14	1.12 (13)
N1—N2—C7—C16	179.53 (7)	C12—C13—C14—C15	0.17 (13)
N2—C7—C8—C9	−0.37 (9)	C11—C10—C15—C14	0.52 (12)
C16—C7—C8—C9	−179.11 (8)	O1—C10—C15—C14	−176.16 (7)
N2—C7—C8—C17	177.70 (7)	C13—C14—C15—C10	−0.97 (13)
C16—C7—C8—C17	−1.05 (14)	C9—C8—C17—O2	178.74 (8)
N2—N1—C9—O1	−174.96 (6)	C7—C8—C17—O2	1.10 (14)

### Hydrogen-bond geometry (Å, °)

**Table:**

*Cg*1 is the centroid of the C1–C6 ring.

**Table:**

*D*—H···*A*	*D*—H	H···*A*	*D*···*A*	*D*—H···*A*
C11—H11A···Cg1^i^	0.95	2.62	3.5052 (8)	156

Symmetry codes: (i) −*x*, −*y*+1, −*z*.
